# Long-Term Follow-Up of Intrastromal Corneal Ring Segments in Paracentral Keratoconus with Coincident Corneal Keratometric, Comatic, and Refractive Axes: Stability of the Procedure

**DOI:** 10.1155/2017/4058026

**Published:** 2017-08-29

**Authors:** Luis Fernández-Vega Cueto, Carlos Lisa, David Madrid-Costa, Jesús Merayo-Lloves, José F. Alfonso

**Affiliations:** ^1^Fernández-Vega Ophthalmological Institute, Oviedo, Spain; ^2^Optics II Department, Optics and Optometry Faculty, Complutense University of Madrid, Madrid, Spain; ^3^Surgery Department, School of Medicine, University of Oviedo, Asturias, Spain

## Abstract

**Purpose:**

To assess the long-term outcomes of implanting intrastromal corneal ring segments (ICRS) in paracentral keratoconic eyes.

**Methods:**

58 eyes with paracentral keratoconus with coincident refractive, keratometric, and comatic axes were evaluated. Uncorrected (UDVA) and corrected (CDVA) distance visual acuity and refractive errors were recorded before and at all follow-up visits. The postoperative follow-up was 5 years. Patients were divided into two groups: group I (30 years old or younger) and group II (more than 30 years old).

**Results:**

The mean UDVA (logMAR) rose from a preoperative 0.83 ± 0.31 to a five-year postoperative 0.42 ± 0.33 (*P* < 0.0001). The mean CDVA varied from 0.16 ± 0.17 to 0.11 ± 0.18 (*P* = 0.0003). Both the UDVA and CDVA were stable over the postoperative period in both groups (*P* > 0.05). The spherical equivalent and the refractive cylinder declined steeply after ICRS implantation in both groups (*P* < 0.001), and were stable over the postoperative period (*P* > 0.05). The keratometric values were also stable over the postoperative follow-up.

**Conclusion:**

Ferrara-type ICRS implantation in keratoconus that meets the characteristics of the sample under study reduces the refractive error at the same time as it improves postoperative UDVA and CDVA six months after surgery and that these results remain stable over five years of follow-up.

## 1. Introduction

Earlier studies have shown that the implantation of intrastromal corneal ring segments (ICRS) is an effective method for the management of keratoconus in terms of refractive and visual outcomes [1–15]. Recently, Ziaei et al. [16] published an interesting review that analysed the outcomes of different reshaping procedures for the management of corneal ectasia. The authors found that all the studies reported a significant reduction in spherical equivalent and keratometric values and an improvement in corrected distance visual acuity (CDVA) after ICRS implantation. In light of this analysis, ICRS implantation seems to be an effective procedure for achieving corneal flattening and improving CDVA. However, there is controversy about the stability of this procedure and whether it is an option for halting the progression of keratoconus. The few studies [2–4, 13, 15] that have been carried out to assess the long-term results of ICRS implantation give different findings with regard to stability. Four of them [3, 4, 13, 15] conclude that the procedure is stable. However, another study found that the procedure was not stable when keratoconus was in progression at the time of surgery [2]. All the studies included keratoconic eyes with different preoperative morphological characteristics.

In this paper, we present the 5-year long-term results of Ferrara-type ICRS implantation using femtosecond laser in 58 paracentral keratoconic eyes with the same preoperative morphological characteristics: paracentral keratoconus with coincident refractive, keratometric, and comatic axes. The aim of this study was to assess whether ICRS implantation in keratoconus patients who meet these preoperative characteristics is an effective, safe, and stable procedure and, therefore, whether these characteristics could be considered as a good prognostic factor for this procedure.

## 2. Patients and Methods

This study was a longitudinal retrospective analysis of the long-term outcomes of Ferrara-type ICRS implantation in eyes with paracentral keratoconus with coincident topographic, refractive, and comatic axes. It was conducted at the Fernández-Vega Ophthalmological Institute, Oviedo, Spain. The tenets of the Declaration of Helsinki were followed, and full ethical approval was obtained from the Institute. After receiving a full explanation of the nature and possible consequences of the study and the surgery, all the patients gave their informed consent.

**Figure 1:**
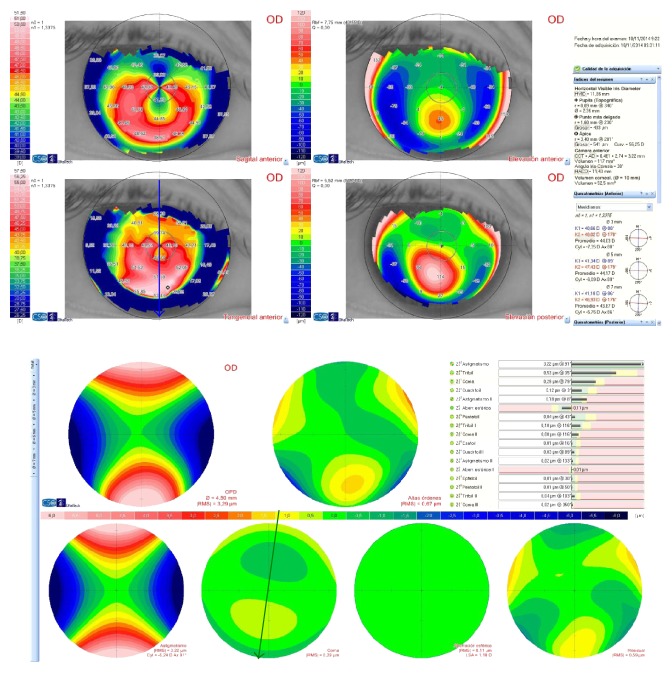
Preoperative corneal topography (Sirius tomographer, CSO, Italy) and coma wavefront map for a 4.5 mm pupil (Sirius tomographer, CSO, Italy). Note the topographic (blue arrow: 88°) and coma (green arrow: 79°) axes.

The criteria required for inclusion in the study were the presence of keratoconus, contact lens intolerance, and a clear cornea, together with a minimum corneal thickness of over 400 *μ*m at the optical zone involved in the implantation (a general criterion for surgery). The Amsler-Krumeich scale was also used to classify keratoconus into stages I and II. Finally, only eyes meeting the following conditions were included (Figure 1):

- The thinnest point on the corneal pachymetry map, charted with an anterior segment optical coherence tomographer (Visante Zeiss-Meditec, Germany), had to be located at a distance of >0.8 mm and ≤1.6 mm from the centre of the pupil, and the thickness at the apex had to be ≥400 *μ*m.
- The differences between the refractive cylinder axis, the flattest corneal meridian measured with a Sirius tomographer (CSO, Italy), and the comatic aberration map, also measured with the Sirius tomographer (CSO, Italy), had to be less than 30°.

The exclusion criteria defined for this study were previous corneal or intraocular surgery, a history of herpetic keratitis, diagnosed autoimmune disease, systemic connective tissue disease, endothelial cell density under 2000 cells/mm^2^, cataract, a history of glaucoma or retinal detachment, macular degeneration or retinopathy, neuro-ophthalmic disease, or a history of ocular inflammation.

Before ICRS implantation, patients had a complete ophthalmologic examination including uncorrected (UDVA) and best-corrected (CDVA) distance visual acuity (ETDRS charts), manifest and cycloplegic refractions, keratometry, corneal topography (Sirius tomographer, CSO, Italy), corneal aberrometry (Sirius tomographer, CSO, Italy), anterior segment optical coherence tomography (Visante Zeiss-Meditec, Germany), endothelial cell count, ultrasonic pachymetry, slit-lamp microscopy, Goldmann applanation tonometry, and binocular indirect ophthalmoscopy. Contact lens use was discontinued 1 month prior to corneal topography. Keratoconus was diagnosed by combining computerised videokeratography of the anterior and posterior corneal surfaces (Sirius tomographer, CSO, Italy), K readings, and corneal pachymetry [17–19]. All the eyes had an inferior-superior corneal shape index greater than 1.40 D (from a mean of 5 points with 30-degree intervals located 3.0 mm from the centre) [20].

Ferrara-type AFR6 ICRS (AJL Ophthalmic, Spain) were implanted in all the eyes studied. These polymethyl methacrylate Ferrara-type ICRS have a triangular cross-section that induces a prismatic effect on the cornea. Their apical diameter is 6.0 mm (flat basis width = 800 *μ*m), with variable thicknesses (150, 200, 250, and 300 *μ*m) and arc lengths (90, 120, 150, and 210 degrees).

The protocol used for ICRS implantation was based on the nomogram developed by Mediphacos Inc. (Keraring Calculation Guidelines 2009; http://smmedical.cl/wpcontent/uploads/2013/10/Agrupado.pdf). The implantation axis of the ICRS was coincident with the flat topographic axis, and ICRS thickness was dependent on the intraoperative pachymetry at the 6 mm implantation zone.

The same surgeon (J.F.A.) performed all the procedures using topical anaesthesia and following our standard procedure previously described [6, 9, 10].

Postoperative treatment consisted of combination antibiotic (tobramycin, 3 mg/ml) and steroid (dexamethasone, 1 mg/ml) eye drops (Tobradex, Alcon Laboratories Inc., Fort Worth, Texas, USA) administered three times daily for 2 weeks, with tapering of the dose over the following 2 weeks.

Postoperative follow-up visits were at 6 months, 1, 3, and 5 years. A standard ophthalmological examination, including slit-lamp biomicroscopy, Goldmann applanation tonometry, binocular indirect ophthalmoscopy, manifest refraction, UDVA and CDVA ETDRS charts, and corneal topography (Sirius tomographer, CSO, Italy), was performed at all follow-up visits. The Thibos and Horner [21] power vector method was used to assess presurgery and postsurgery refraction findings.

Given that keratoconus usually progresses until the patients reach their thirties, when it normally begins to stabilise [20], analyses of the long-term outcomes were done in two groups. Patients in group I were 30 years old or younger at the time of ICRS implantation, and those in group II were more than 30 years old when the surgery was performed.

Data analysis was performed with SPSS for Windows software (version 15.0, SPSS, Inc.). Normality was checked using the Kolmogorov-Smirnov test, and outcomes were compared using *t*-tests and analysis of variance with multiple comparison. A *P* value of less than 0.05 was regarded as proof of statistical significance. Data are shown as the mean ± SD.

## 3. Results

The study was carried out with 58 keratoconic eyes from 51 patients.  Table 1 shows patient demographics. ICRS were successfully implanted in all 58 eyes studied in this series, with no intra- or postoperative complications.

**Figure 2:**
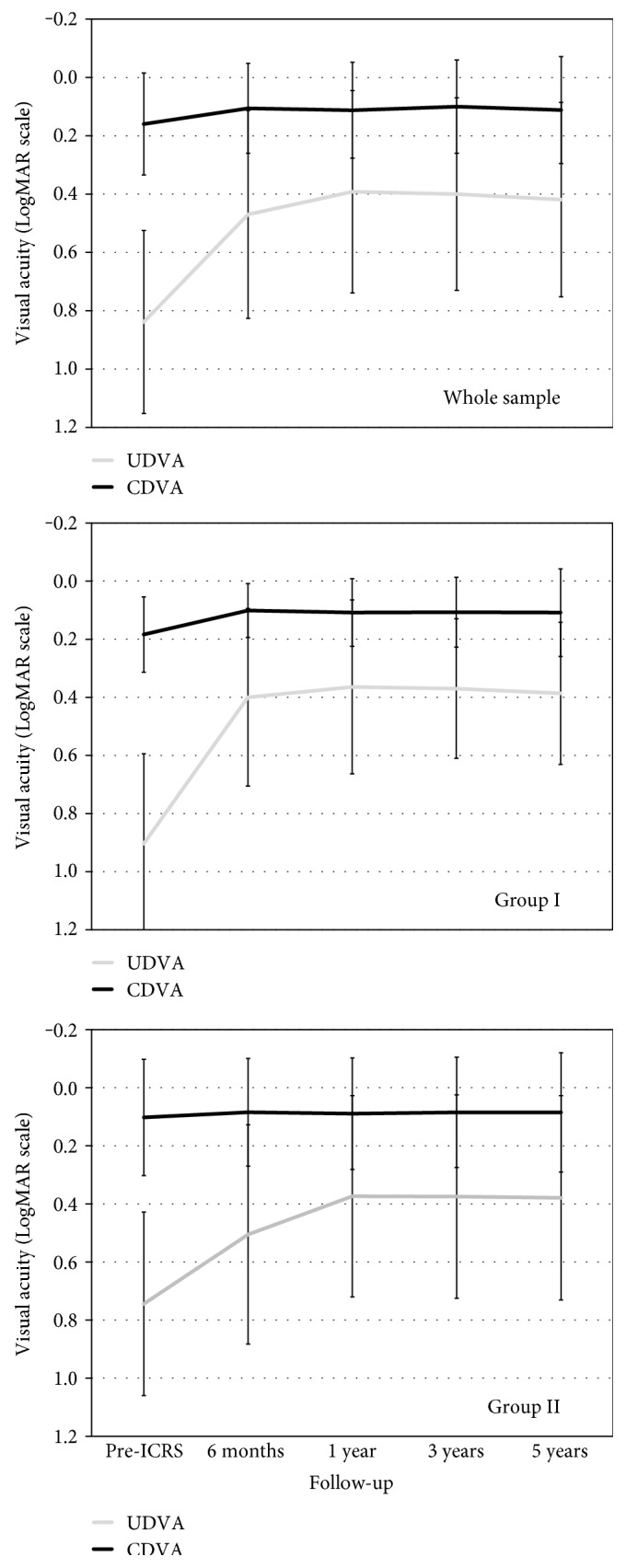
Uncorrected visual acuity (UDVA) and corrected distance visual acuity (CDVA) before ICRS and over the postoperative period for the whole sample and for group I (younger patients) and group II (older patients).

**Figure 3:**
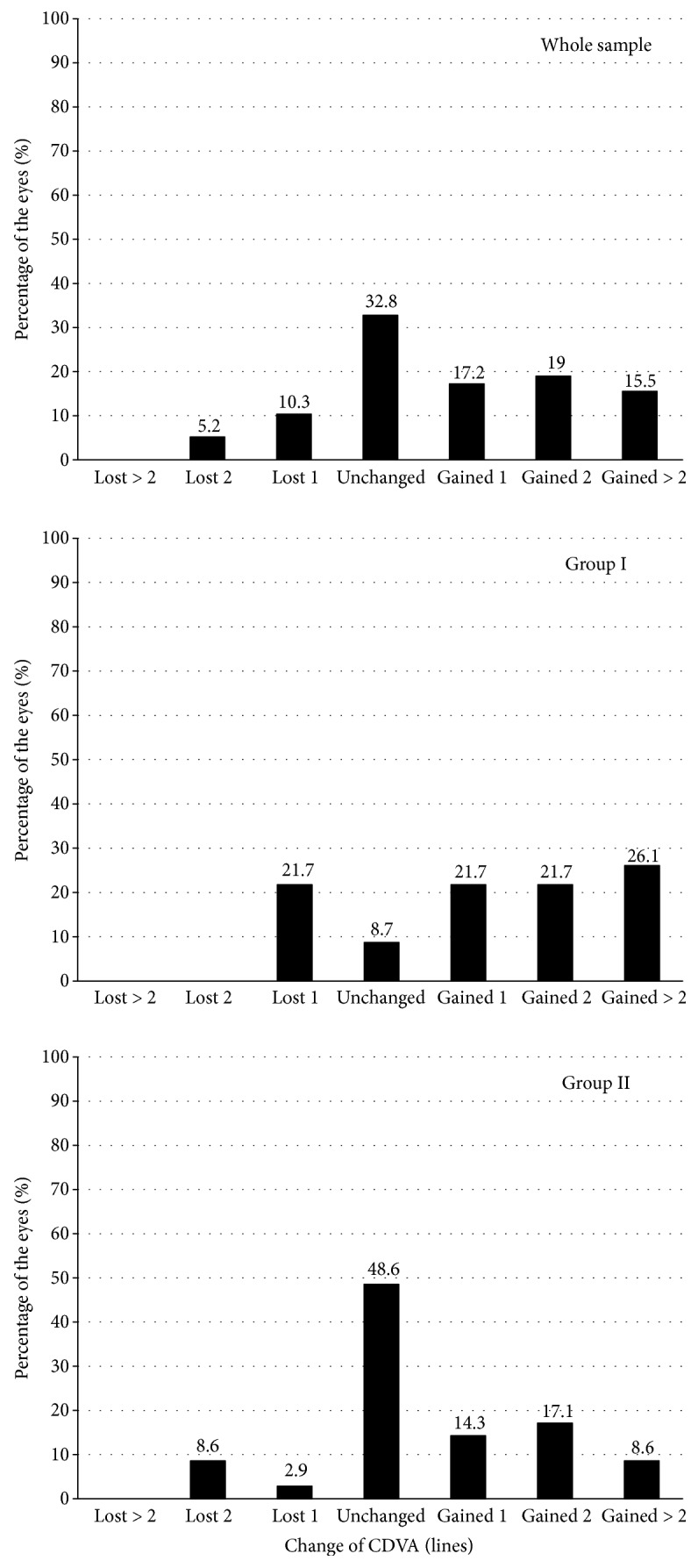
Variation in CDVA five years after ICRS implantation (safety) for the whole sample and for group I (younger patients) and group II (older patients).

Figure 2 shows the UDVA and CDVA values before implantation and over the postoperative period. The mean UDVA (logMAR scale) rose from a preoperative 0.83 ± 0.31 to a five-year postoperative 0.42 ± 0.33 (*P* < 0.0001). The mean CDVA varied in turn from 0.16 ± 0.17 to 0.11 ± 0.18 (*P* = 0.0003). Both the UDVA and CDVA were stable over the postoperative period in both groups (*P* > 0.05) (Figure 2). The five-year efficacy index (mean postoperative UDVA/mean preoperative CDVA) was 0.65 for the whole sample, and 0.69 and 0.64 for groups I and II, respectively, while the five-year safety index (ratio of postoperative to preoperative monocular CDVA) for ICRS implantation was 1.12 for the whole sample, and 1.2 and 1.05 for groups I and II, respectively. Five years after surgery, none of the eyes had lost more than 2 lines of CDVA compared to preoperative values. Only three eyes (5.2%) lost 2 lines of CDVA, six eyes (10.3%) showed a decrease of 1 line, and the rest of the eyes (84.5%) maintained or improved their CDVA compared to preoperative values (Figure 3).

**Figure 4:**
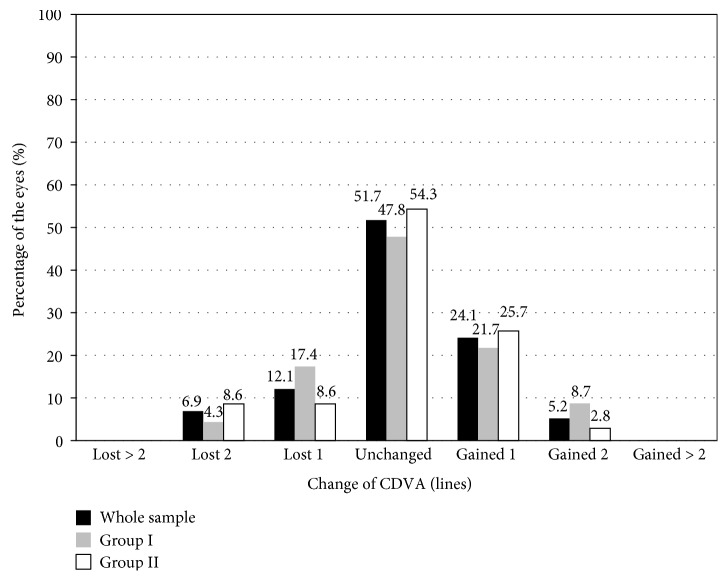
Variation in CDVA between the visit at 6 months and the final visit at five years, for the whole sample and for group I (younger patients) and group II (older patients).

In order to study the stability of the surgery in terms of CDVA, we examined the changes in CDVA over the postoperative period (Figure 4). None of the eyes had a decrease of more than two lines of CDVA over the postoperative follow-up period (Figure 4). Between the visit at 6 months and the final visit at five years, only four eyes had lost 2 lines of CDVA (one eye from group I and three eyes from group II), and seven eyes (four from group I and three from group II) had lost 1 line of CDVA. In the rest of the eyes, the CDVA values were maintained or improved (Figure 4).

**Figure 5:**
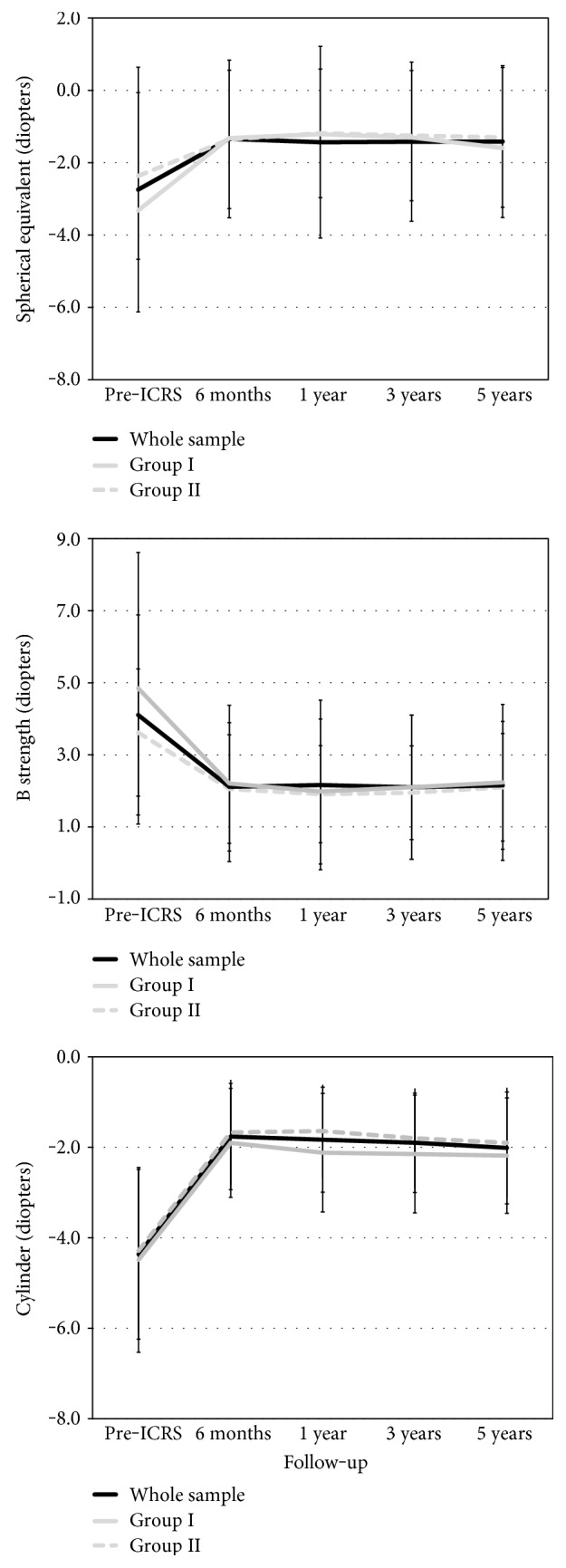
Spherical equivalent, the blur strength value (B), and refractive cylinder before ICRS and over the postoperative period for the whole sample and for group I (younger patients) and group II (older patients).

The spherical equivalent declined from a preoperative −2.74 ± 3.38 D to a five-year postoperative value of −1.42 ± 2.10 D (*P* < 0.001), and the blur strength value (B) dropped from 4.10 ± 2.77 D to 2.15 ± 1.77 D (*P* < 0.001). Both the spherical equivalent and *B* value were stable over the postoperative period in both groups (*P* > 0.05) (Figure 5). The refractive cylinder changed from −4.37 ± 1.87 D preoperatively to −2.01 ± 1.24 D five years after ICRS implantation (*P* < 0.001). The refractive cylinder was also stable over the postoperative period in both groups (*P* > 0.05). Between the 6-month and 1-year visits, the change in the mean cylinder was −0.21 ± 0.78 D and 0.02 ± 0.88 D for groups I and II, respectively (Figure 5).

**Figure 6:**
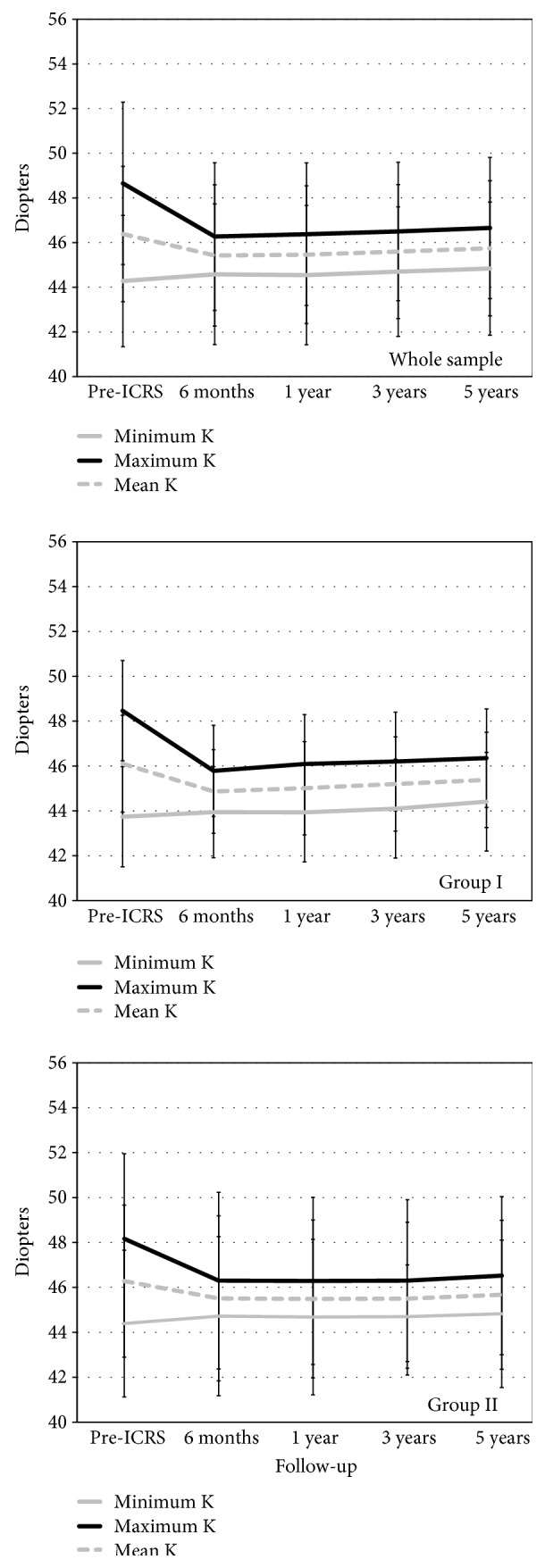
The keratometric values before ICRS and over the postoperative period for the whole sample and for group I (younger patients) and group II (older patients).

The keratometric values, both preoperatively and over the follow-up period, are shown in Figure 6. The mean maximum and minimum keratometric values were stable in both groups over the postoperative follow-up (*P* > 0.05).

## 4. Discussion

To date, there are several studies that have evaluated the outcomes of ICRS implantation for the treatment of keratoconic patients [1–15]. However, few studies have spanned [2–4, 13, 15] five or more years to allow an assessment of the long-term clinical and refractive results and therefore the stability of the procedure. Kymionis et al. [15] reported long-term (five years) outcomes of Intacs implantation in 17 eyes with keratoconus. The results of this study showed that Intacs ICRS implantation improves visual acuity, refraction, and topographic findings in keratoconic patients six months after surgery and that these remain stable over the follow-up period.

Long-term results of Ferrara-type ICRS implantation have been reported by Torquetti et al. in two studies [3, 13], one with a five-year follow-up [13] and the other with a ten-year follow-up [3]; they found that the refractive and visual outcomes were stable over the follow-up period. Although “progression of keratoconus” was an inclusion criterion in both studies, the mean age of the patients at the time of surgery was 39 years, and it is known that the progression of keratoconus tends to cease as patients reach their thirties. Consequently, it is difficult to ascertain whether progression is halted by the effect of ICRS or by age. Either way, these results suggest that the procedure is stable. Vega-Estrada et al. [2, 4] carried out two studies in which they analysed the five-year long-term effects of ICRS implantation in both nonprogressive keratoconus [4] and progressive keratoconus [2]. The authors concluded from the first study that the changes induced by ICRS are stable over a long period in patients with no evidence of keratoconus progression at the time of surgery [4]. In their second study [2], they examined the outcomes of ICRS implantation in young patients showing evidence of keratoconus progression and found that although ICRS implantation improved the visual and refractive outcomes in the short term, there was regression in the long term, which suggests that this procedure is not stable in young patients with evidence of keratoconus progression. However, it is important to note that this study had certain limitations. It was carried out on a total of 18 eyes, of which 13 eyes were implanted with Intacs ICRS (10 with the mechanical procedure and 3 with femtosecond) and 5 eyes were implanted with Ferrara-type ICRS (4 with femtosecond technology and 1 with the mechanical procedure). In addition, the keratoconus included in this study showed very strong progression of the disease (the mean K reading increased 3.17 D and the mean spherical equivalent 1.86 D in 6 months immediately prior to surgery). In any case, it would be appropriate to conduct further studies because, as the authors suggest, if these results are confirmed, combining corneal cross-linking with ICRS implantation for remodelling corneal shape and halting the progression of keratoconus in this type of patient could be very interesting.

In the current study, we analysed the visual and refractive outcomes of ICRS implantation in 58 paracentral keratoconus with the same preoperative characteristics over a five-year follow-up period. Our results show an increase in UDVA and CDVA values and a decrease in spherical equivalent, *B* value, and maximum keratometric values six months after ICRS implantation. These parameters remained without significant changes over the five-year follow-up period. As we did not consider evidence of keratoconus progression as a criterion for inclusion in the study, we do not know if the keratoconus was in progression at the time of surgery. However, it is well established that the progression of keratoconus is more acute up to the third decade of life [20]. For this reason, we divided the patients into two groups in order to analyse the results. Group I was composed of patients who were 30 years old or younger at the time of surgery and group II of patients who were older than 30 years. On analysing the outcomes in both groups, it was observed that there was an improvement after surgery in all the parameters studied, both refractive and visual (UDVA, CDVA, spherical equivalent, the blur strength (B) value, the refractive cylinder, and keratometric values), and that these were stable in both groups over the postoperative follow-up period. None of the eyes showed a decrease of more than two lines of CDVA over the postoperative follow-up period. Between the 6-month visit and the final visit, only four eyes lost 2 lines of CDVA, and of these four, just one was in group I (younger patients), that is, when the risk of keratoconus progression could be considered higher. In addition, the change in the mean cylinder between the 6-month and 1-year visits was −0.21 ± 0.78 D and 0.02 ± 0.88 D for groups I and II, respectively. Similarly, the mean maximum and minimum keratometric values were stable over the postoperative follow-up period in both groups.

As we pointed out before, we cannot conclude that the procedure slows down keratoconus progression. What we can confirm, however, is that the Ferrara-type ICRS implantation using Femtosecond laser is a safe, effective, and stable procedure in nonprogressive paracentral keratoconus with coincident refractive, keratometric, and comatic axes, even in young patients where the risk of keratoconus progression over the follow-up period is higher. It is important to bear in mind that the keratoconus in this study had the same preoperative morphological characteristics (paracentral location and coincident refractive, keratometric, and comatic axes). We should therefore proceed with caution because, in terms of stability, different results could be obtained from patients with keratoconus that does not have these preoperative characteristics. A previous study found that better visual acuity results were obtained in keratoconic eyes with coincident axes [22]. Therefore, both studies could suggest that keratoconus with coincident refractive, keratometric, and comatic axes could have a good prognostic indicator for ICRS implantation.

These results ultimately suggest that Ferrara-type implantation in keratoconus that meets the morphological characteristics of the sample under study is a stable procedure over five years of follow-up.

## Figures and Tables

**Table 1:** Patients' demographics: age, pre-ICRS implantation spherical equivalent (SE), manifest refraction (refractive sphere and cylinder), undistance visual acuity (UDVA) (logMAR scale), corrected distance visual acuity (CDVA) (logMAR scale) pre-keratometry (K) value, and root mean square (RMS) for coma-like aberration shown as mean ± standard deviation (SD) and range.

Characteristic	Whole sample	Group I (younger patients)	Group II (older patients)
Eyes (*n*)	58	23	35
Age (years)	34 ± 9.2	25.35 ± 3.88	40.23 ± 6.56^∗^
Mean SE (D)	−2.74 ± 3.38	−3.32 ± 4.57	−2.36 ± 2.30
Mean refractive sphere (D)	−0.50 ± 3.24	−1.08 ± 4.45	−0.22 ± 2.11
Mean refractive cylinder (D)	−4.36 ± 1.87	−4.49 ± 2.02	−4.28 ± 1.78
UDVA (logMAR)	0.83 ± 0.31	0.90 ± 0.31	0.74 ± 0.32
CDVA (logMAR)	0.16 ± 0.17	0.18 ± 0.13	0.10 ± 0.20
Mean K minimum (D)	44.41 ± 3.08	44.00 ± 2.52	44.62 ± 3.47
Range K minimum (D)	39.25 to 53	40 to 49.75	39.25 to 53
Mean K maximum (D)	48.65 ± 3.63	48.79 ± 2.84	48.46 ± 4.11
Range K maximum (D)	42.50 to 58	44.25 to 55.5	42.50 to 58
RMS for coma-like for 4.5 mm of pupil size (*μ*m)	1.03 ± 0.65	0.96 ± 0.65	1.12 ± 0.60
Range RMS for coma-like (*μ*m)	0.1 to 2.23	0.1 to 2.23	0.1 to 2.08

^∗^Statistically significant between groups.
